# The psychological distress of gastrointestinal cancer patients and its association with quality of life among different genders

**DOI:** 10.1007/s00520-024-08533-z

**Published:** 2024-05-06

**Authors:** Qinqin Cheng, Jianfei Xie, Yinglong Duan, Juan Li, Zhengdi She, Wen Lu, Yongyi Chen

**Affiliations:** 1 Hunan Cancer Hospital, Changsha, Hunan 410013 People’s Republic of China; 2 Nursing Department, The Third Xiangya Hospital, Central South University, Changsha, Hunan 410013 People’s Republic of China

**Keywords:** Psychological distress, Gastrointestinal neoplasms, Quality of life, Genders, Association

## Abstract

**Background:**

Psychological distress is a prevalent unpleasant experience faced by many cancer patients. However, the psychological distress among gastrointestinal (GI) cancer patients is scarcely explored. Moreover, the association between psychological distress and quality of life in different genders has yet to be explored.

**Aims:**

To explore the psychological distress among GI cancer patients and examine its association with quality of life among different genders.

**Methods:**

This study was a cross-sectional study. A total of 237 gastrointestinal cancer patients completed the distress thermometer and the Functional Assessment of Chronic Illness Therapy-General.

**Results:**

The mean score of psychological distress of the participants was 3.04 (SD = 2.90). A greater proportion of female gastrointestinal cancer patients (52.8%) had clinically relevant psychological distress compared to males (35.9%). The quality of life was negatively associated with their psychological distress (B =  − 1.502, 95%CI: − 2.759 to − 0.245, *p* = 0.019) among gastrointestinal cancer patients. Such association was stronger among males compared to females in gastrointestinal cancer patients (Interaction term, B =  − 1.713, 95%CI: − 3.123 to − 0.303, *p* = 0.017).

**Conclusions:**

These findings suggest that healthcare providers should attach their attention to gastrointestinal cancer patients’ psychological distress, especially females. Longitudinal studies could adopted to track the changes in psychological distress and its association with quality of life over time among different genders. In future intervention studies, the focus of psychological interventions needs to be gender-specific.

## Introduction

Gastrointestinal (GI) cancer is a term for the group of cancers that affect the digestive system, which includes esophageal cancer, stomach cancer, colorectal cancer, pancreatic cancer, liver cancer, etc. [1]. In China, GI cancers, including liver cancer, esophageal cancer, and gastric cancer, created a massive burden of cancer incidence, with 1.21 million newly diagnosed cases in 2020 (two-thirds of the world’s total) [2]. In spite of their high incidence, the mortality of GI cancers is relatively high due to poor prognosis and late-stage manifestation[3]. GI cancers accounted for 45% of all cancer deaths in 2020[3]. The poor prognosis, treatment-related side effects and burden often lead GI cancer patients to suffer from negative emotions [4].

Psychological distress is defined as an unpleasant experience that can make the patient have difficulty coping with the disease, the symptoms, or the treatment [5]. It is prevalent at different time points in fighting the illness among cancer patients. Measured by the distress thermometer, psychological distress is suffered by 25.3% to 71.7% of breast cancer patients [6], 49.04% of lung cancer [7], and 35% to 91% of patients with different cancer types [8–10]. An increasing emphasis is placed on the psychological distress experienced by cancer populations. However, limited evidence on psychological distress among GI cancer patients exists. Previous research notes that psychological distress not only brings unpleasant experiences but also further impacts the patients’ treatment compliance and outcomes [11]. Therefore, early recognition and identification of psychological distress are vital for helping GI patients to survive their disease journey.

Previous studies also have investigated the association between psychological distress and quality of life among cancer patients. For example, a descriptive cross-sectional study found that psychological distress was correlated with the quality of life among breast cancer patients [12]. Similar results were also observed among other cancer groups [13–17]. Therefore, the existing studies revealed the negative association between psychological distress and quality of life among cancer patients.

With the existing evidence on the relationship between psychological distress and quality of life, there is no research to investigate whether there are gender differences in such relationships. Whether gender and psychological distress have interactions on association with the quality of life remains unknown and is warranted to be explored. However, there is reason to hypothesize gender differences in the relationship between psychological distress and quality of life. For instance, males and females have different responses and actions when facing psychological distress [18]. The varying reactions may have an influence on the association between psychological distress and quality of life. Females usually are more likely to externalize emotional expressions [19]. Therefore, it is hypothesized that the impact of psychological distress on quality of life is more severe in males.

In light of the aforementioned research gaps, this study aimed to explore the psychological distress among GI cancer patients and examine its association with quality of life among different genders. We hypothesized that psychological distress among GI cancer patients was prevalent and it was negatively associated with the quality of life. Furthermore, the association was stronger in males.

## Methods

A descriptive, correlational cross-sectional study was conducted. This study was approved by the Institutional Review Board at Hunan Cancer Hospital (Approval Number: SBQLL-2020–141) and conducted in accordance with the Declaration of Helsinki. The report of this study conformed to the STrengthening the Reporting of OBservational studies in Epidemiology.

### Participants

In this study, GI patients were recruited by convenience sampling between May and October 2022 from a tertiary cancer hospital in the Chinese Mainland. This tertiary cancer hospital is a provincial public hospital that serves cancer patients from both the rural and urban areas in this province. GI cancer patients who were 18 or above years old, receiving treatments, informed of their illness, and capable of reading and understanding the questions were invited to participate in this study. However, those with mental impairments judged by the physicians and participating in other psychological interventions were excluded.

## Measures

### Sociodemographic and clinical characteristics

A structured information questionnaire was used to measure the participants’ sociodemographic characteristics (i.e. age, gender, education level, marital status, occupation) and clinical characteristics (diagnosis, stage of cancer, time since diagnosis and whether having other diseases).

### Psychological distress

Participants’ psychological distress was measured by the distress thermometer. The distress thermometer is a self-reported instrument used to assess the respondents’ extent of psychological distress from the previous week. It includes one single item being responded to from 0 (no distress) to 10 (extreme distress). A higher score indicates more severe psychological distress. The cut-off score of 4 indicates clinically relevant psychological distress. The Chinese version of the distress thermometer was validated in 4815 cancer patients and showed its appropriateness among Chinese cancer patients [20]. The distress thermometer showed good discriminating accuracy, and the sensitivity and specificity for a cut-off of 4 were 0.81 and 0.88, respectively [21].

### Functional Assessment of Chronic Illness Therapy-General

Functional Assessment of Chronic Illness Therapy-General (FACT-G) is a 27-item scale used to measure the quality of life of cancer patients. It consists of four dimensions: physical, social, emotional and functional wellbeing. Each item was responded to with a five-point Likert scale, ranging from ‘0 = not at all’ to ‘4 = very much’. The total score ranges from 0 to 108, with a higher score indicating a better quality of life [22]. The Chinese version demonstrated had the same four dimensions as the original version. The Cronbach α coefficients were above 0.8 for the four dimensions, indicating that the Chinese version of FCAT-G had good reliability [23]. The factor analysis demonstrated that the four factors accounted for 65.8% of the total variance, representing good construct validity [23].

### Data collection procedure

The data was collected by research assistants, who had received training on how to collect data using questionnaires. The research assistants approached the eligible participants and invited them to participate in this study a week after their admission. All the participants were provided with comprehensive information about this study, including the purpose, potential benefits, or harms of the study. Sufficient time was given to the participants to ask any questions they had regarding the study, enabling them to make an informed decision about their participation. Participating in this study was totally voluntary, and declining to participate would not have any impact on their treatment or care. Once the participants provided their consent to participate, they independently completed the questionnaires. In case of any inquiries or doubts about the questionnaire items, the research assistants were available to provide explanations and assistance.

### Data analysis

We used R software version 4.3.0 to analyze the data. The sociodemographic and clinical characteristics, as well as the participants’ psychological distress and quality of life, were summarized using appropriate descriptive statistics, including means (standard deviations) for normative continuous data or median (inter‐quartile range) for skewed data and n(%) for categorical data. Univariable analyses, i.e., Pearson correlation, t-test, and analysis of variance, were conducted to explore the association between the sociodemographic and clinical characteristics and quality of life. In order to explore the association between psychological distress and quality of life, multiple linear regression analysis was conducted with quality of life as the independent variable using the entry method. The sociodemographic and clinical characteristics that showed significant association (*p* < 0.25) with quality of life in univariable analysis were adjusted. In addition, interaction terms were used to investigate whether the association between psychological distress and quality of life differed in terms of gender. *P*-values below 0.05 were regarded as statistically significant, with the exception of the interaction analyses, which employed *p*-values below 0.10.

## Results

### Sociodemographic characteristics of the participants

A total of 237 GI cancer patients participated in this study. The participants’ mean age was 54.35 (SD = 10.54). Most of the participants were male (n = 184, 77.6%), married (n = 223, 94.1%), farmers (n = 99, 41.8%), had an education level of junior high school (n = 84, 35.4%), and lived in the rural area (n = 164, 69.2%). Regarding the clinical related characteristics, 176 participants (74.3%) were diagnosed with liver cancer and 169 (71.3%)were at Stage III or above. About half of them (n = 131, 55.2%) had a disease course with cancer less than 6 months. The majority of them (n = 181, 76.4) had other chronic diseases.

### Psychological distress of GI cancer patients

**Fig. 1:**
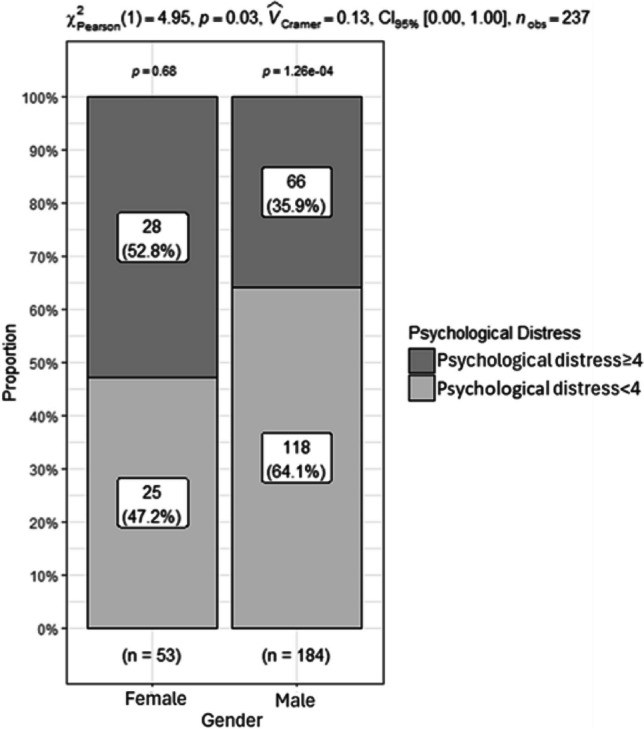
Proportion of psychological distress ≥ 4 in males and females

The mean score of psychological distress of the participants was 3.04 (SD = 2.90). Among all the 237 participants, 94 (39.7%) participants had clinically relevant psychological distress (score ≥ 4). Among males, 66 (35.9%) participants had clinically relevant psychological distress (score ≥ 4), whereas among females, 28 (52.8%) had clinically relevant psychological distress (score ≥ 4). As shown in Fig. 1, the proportion of participants with clinically relevant psychological distress differed significantly between males and females (*p* = 0.026).

### The association between psychological distress and quality of life

**Fig. 2:**
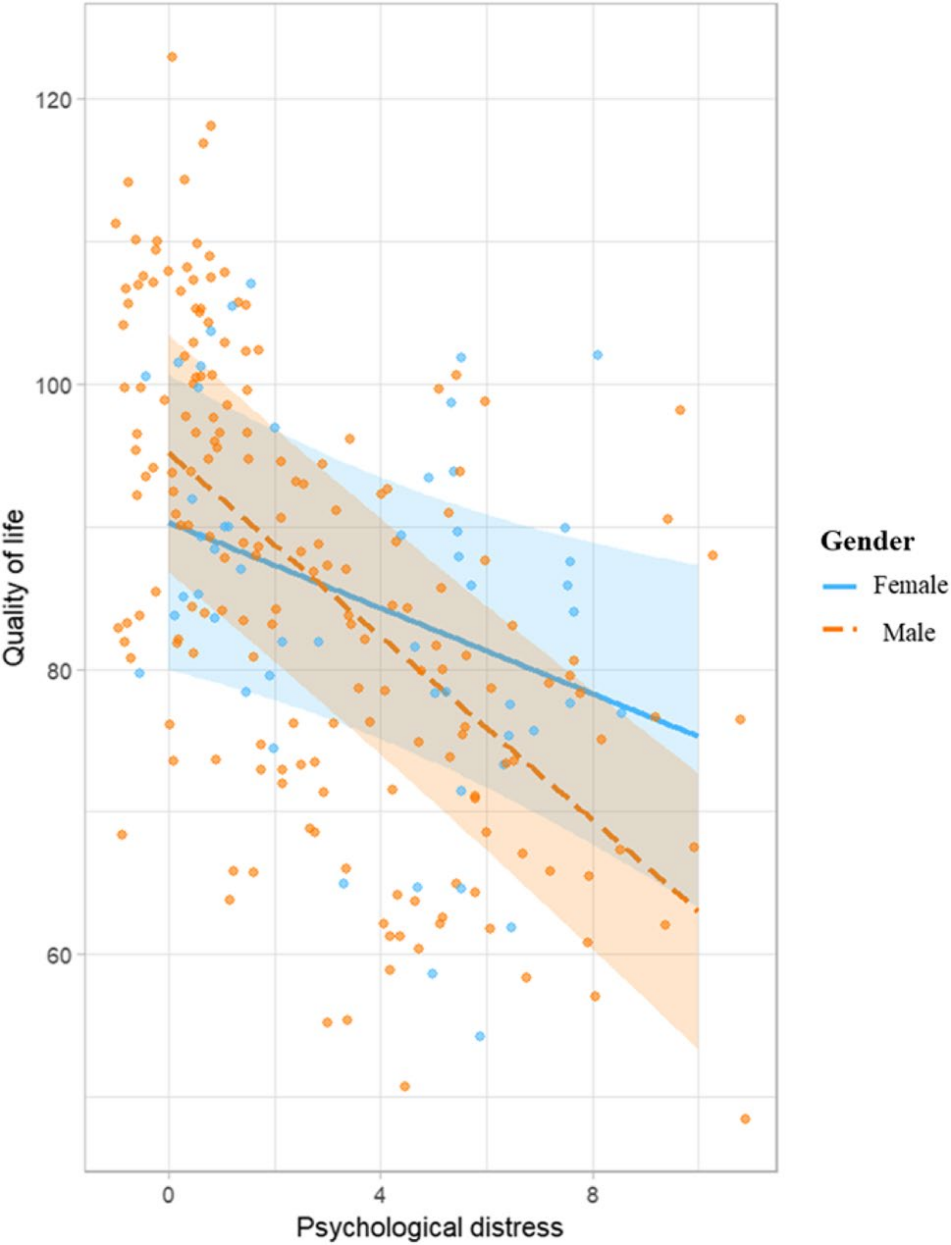
The association between psychological distress and quality of life among different genders

The univariable analysis results showed that the variables, namely, education, marital status, residence, occupation, diagnosis, stage of cancer, and whether having other chronic diseases, were associated with quality of life at* p* < 0.25. Therefore, these variables were adjusted when conducting the linear regression analysis. As shown in Table 1, model 0 was the linear regression model between the quality of life and these sociodemographic and clinical characteristics. Model 1 was conducted, including gender, psychological distress, and their interaction effect, by adjusting the above variables. The results showed that the GI cancer patients’ quality of life was negatively associated with their psychological distress (B =  − 1.502, 95%CI: − 2.759 to − 0.245, *p* = 0.019). The associations between psychological distress and quality of life showed significant interaction with gender (B =  − 1.713, 95%CI: − 3.123 to − 0.303, *p* = 0.017). In males, psychological distress had a greater association with the quality of life. The gender differences in the association between psychological distress and quality of life are presented in Fig. 2.

**Table 1:** The association between psychological distress and quality of life

Variables	*B*	95%CI	SE	*t*	*p*-value
Model 0					
Education					
Primary school or below (Ref)					
Junior high school	− 0.883	− 6.507, 4.742	2.854	− 0.309	0.757
Senior high school	1.472	− 5.088, 8.033	3.329	0.442	0.659
College or above	4.781	− 3.089, 12.651	3.994	1.197	0.233
Marital status					
Married (Ref)					
Unmarried/divorced/widowed	− 10.043	− 18.668, − 1.418	4.377	− 2.295	0.023
Residence					
Rural (Ref)					
Urban	6.196	1.1366, 11.255	2.567	2.414	0.017
Occupation					
Employees/self-employed entrepreneurs (Ref)					
Farmers	0.530	− 5.636, 6.696	3.129	0.169	0.866
Retired/unemployed	− 5.987	− 11.783, − 0.191	2.941	− 2.036	0.043
Other	− 7.160	− 15.711, 1.390	4.339	− 1.650	0.100
Diagnosis					
Liver cancer (Ref)					
Colorectal cancer	6.992	− 0.735, 14.718	3.920	1.783	0.0759
Bile duct cancer	− 3.642	− 11.346, 4.062	3.909	− 0.932	0.353
Other	− 7.960	− 18.046, 2.127	5.118	− 1.555	0.121
Stage of cancer					
Stage I (Ref)					
Stage II	− 12.950	− 21.770, − 4.130	4.475	− 2.894	0.004
Stage III	− 11.292	− 19.303, − 3.280	4.065	− 2.778	0.006
Stage IV	− 15.772	− 25.966, − 5.578	5.172	− 3.049	0.003
Not clear	− 6.133	− 20.946, 8.681	7.517	− 0.816	0.415
Whether having other chronic diseases					
No (Ref)					
Yes	− 8.569	− 13.435, − 3.703	2.4690	− 3.471	< 0.001
Model 1					
Gender					
Female (Ref)					
Male	4.868	− 2.001, 11.736	3.485	1.397	0.164
Psychological distress	− 1.502	− 2.759, − 0.245	0.638	− 2.355	0.019
Gender and psychological distress	− 1.713	− 3.123, − 0.303	0.715	− 2.395	0.017

*Ref* reference category of categorical variable

*B* unstadardised coefficient, *CI* confident interval, *SE* standard error

Model 0: adjusted *R*^2^ = 0. 136, *F* = 3.312, *p* < 0.001; model 1: adjusted *R*^2^ = 0.388, *F* = 8.865, *p* < 0.001

ANOVA (model 0, model1): *F* = 31.208, *p* < 0.001

## Discussion

This study investigated the psychological distress among GI cancer patients and explored its association with quality of life among different genders. This kind of knowledge could contribute to a better understanding of the association between psychological distress and quality of life and inform healthcare providers of specific attention to which gender.

Current evidence on the prevalence of psychological distress among different cancer patients is inconsistent. This study found that 94 (39.7%) participants had clinically relevant psychological distress (score ≥ 4). This prevalence was lower than those reported in some studies [24, 25] but higher than other studies conducted in China using distress thermometer [26, 27]. These differences may be related to the variations in cancer type and study populations in different contexts. For example, Hong et al. reported that the prevalence of clinically significant psychological distress was 76.97% among newly diagnosed gastric cancer [25]. As psychological distress was more prominent among newly diagnosed cancer patients [28], the prevalence of psychological distress reported by Hong et al. was much higher than that of our study. In this study, we investigated GI cancer patients receiving treatment in inpatient wards. The relatively high prevalence of psychological distress indicates that healthcare providers should attach their attention to the psychological distress of GI cancer patients.

Moreover, we found that the proportion of GI patients with significant psychological distress was higher in females than in males. This finding was similar to other studies [29, 30]. This may be attributed to the fact that when facing difficult situations, men usually present more positive attitudes and higher psychological flexibility, which were related to less psychological distress [31]. In contrast, women demonstrated more depressive symptoms, anxiety, and functional limitations than men [32]. This result implies that female GI cancer patients are more vulnerable to psychological distress, and healthcare professionals need to pay more attention to female GI cancer patients.

This study also found that the GI cancer patients’ quality of life was negatively associated with their psychosocial distress. Those GI cancer patients who had higher levels of psychological distress were more likely to have a worse quality of life. This result confirmed similar findings in previous studies [16, 17, 25]. Furthermore, this study revealed variations in the link between psychological distress and quality of life across different genders. Among GI cancer patients, males exhibited a stronger negative association between psychological distress and quality of life compared to females. This gender-based difference could be attributed to several plausible explanations. First, women are more likely to have stronger social support networks and tend to seek support from others [33]. Strong social support can buffer against the negative effects of psychological distress on their quality of life [34]. Conversely, males often suppress their negative emotions, even when experiencing psychological distress, thereby exacerbating the impact on their quality of life [35]. Additionally, individual differences in how men and women perceive and process psychological distress may also contribute to this difference. Characteristics such as personality traits, resilience, and cognitive patterns might vary between genders [36]. These differences may also influence the associations between psychological distress and quality of life. Apart from these psychosocial factors, biological differences exist between different sexes [37]. Hormonal variations can influence stress responses and coping mechanisms when facing challenging situations [18]. Recent research has found that women may have a greater stress response than men [38]. The divergent responses to stress might contribute to variations in the way psychological distress is associated with quality of life. However, it is crucial to recognize that the underlying reasons for the observed gender differences in the association between psychological distress and quality of life among GI cancer patients are likely to be complex and multifaceted. Further research is needed to explore these factors more deeply and provide a clearer understanding of the mechanisms underlying this gender-based disparity.

The study highlights a paradox where male GI cancer patients might have a lower prevalence of clinically significant psychological distress compared to other groups, yet the distress they experience has a stronger association with their overall quality of life. These findings indicate that healthcare providers should be aware of this disparity and the potential for psychological distress to affect male patients’ quality of life disproportionately. Consequently, it becomes imperative for healthcare professionals to identify clinically significant psychological distress among male GI cancer patients. When such distress is present, healthcare services specifically targeting psychological distress should be considered to address and alleviate its impact on their quality of life.

## Implications

In this study, we found that the psychological distress among GI cancer patients was pretty high. Their psychological distress was negatively associated with quality of life. Further, we found that this kind of association is stronger among male GI cancer patients. The findings of this study had specific implications for clinical practice. First, given the relatively high prevalence of psychological distress, due attention should be paid to this population. Routine screening for psychological distress needs to be incorporated into the care of GI cancer patients. Then, timely support can be provided by healthcare providers. Second, healthcare providers need to pay more attention to GI cancer patients who are more vulnerable to psychological distress. In this study, the significantly higher proportion of female GI cancer patients with psychological distress emphasizes the need for healthcare providers to recognize the specific vulnerabilities and challenges faced by female GI cancer patients. Third, the discrepancy of association between psychological distress and quality of life implies that healthcare providers need to address the psychological distress of GI cancer patients of different genders appropriately. For females, interventions may need to address specific stressors that contribute to high psychological distress, such as body image issues, fear of recurrence, or social support needs. For males, healthcare providers may need to focus on the potential underlying reasons that may lead to a strong association between psychological distress and quality of life. Recognizing that male patients may hide their distress, healthcare providers should create a safe and non-judgmental environment to encourage emotional expression. Additionally, strategies, such as providing information or education about the possible impact of psychological distress on their quality of life, are essential to prompt them to promote their active coping and seek available support.

## Strengths and limitations

This study had several strengths. To our best knowledge, this study was the first of its kind to explore the psychological distress among GI cancer patients and explore its associations with quality of life among different genders. The findings contributed to the existing body of knowledge on psychological distress in GI cancer patients and enhanced our understanding of how psychological distress is associated with quality of life among individuals of different genders.

Despite its strengths, it is important to acknowledge the limitations of this study. Firstly, all the participants in this study were recruited from a single hospital using convenience sampling, which may lead to selection bias and limit the generalizability of the findings. To improve the external validity of future research, it is crucial to recruit participants from multiple sources using more diverse sampling methods, such as complex or multi-stage sampling[39]. Secondly, it is worth noting that the majority of the study sample consisted of males (77.6%), reflecting the predominance of males among GI cancer patients. Future studies should include a balanced representation of males and females in their samples to verify the results.

## Conclusions

Almost 40% of GI cancer patients had clinically relevant psychological distress. More female GI cancer patients (52.8%) had psychological distress than males (35.9%). Their psychological distress was negatively associated with quality of life. Moreover, this kind of association was stronger in male GI cancer patients. Longitudinal studies could be adopted to track the changes in psychological distress and its association with quality of life over time among different genders. In future intervention studies, it is suggested that psychological interventions be developed with consideration of gender differences.

Supplementary information.

## Data Availability

Data could be provided by the corresponding author on a reasonable request.

## References

[1] Costa NR, Gil da Costa RM, Medeiros R (2018). A viral map of gastrointestinal cancers. Life Sci.

[2] Wild C, Weiderpass E, Stewart BW (2020). World cancer report: cancer research for cancer prevention.

[3] Cao W, Chen HD, Yu YW, Li N, Chen WQ (2021). Changing profiles of cancer burden worldwide and in China: a secondary analysis of the global cancer statistics 2020. Chin Med J (Engl).

[4] Chung J, Ju G, Yang J, Jeong J, Jeong Y, Choi MK, Kwon J, Lee KH, Kim ST, Han HS (2018). Prevalence of and factors associated with anxiety and depression in Korean patients with newly diagnosed advanced gastrointestinal cancer. Korean J Intern Med.

[5] National Comprehensive Cancer Network (2022) Distress Management. https://www.nccn.org/professionals/physician_gls/pdf/distress.pdf. Accessed 23 August 2022

[6] Sun H, Lv H, Zeng H, Niu L, Yan M (2022). Distress thermometer in breast cancer: systematic review and meta-analysis. BMJ Support Palliat Care.

[7] Zhang L, Liu X, Tong F, Zou R, Peng W, Yang H, Huang X, Yi L, Wen M, Jiang L, Liu F (2022). Lung cancer distress: screening thermometer meta-analysis. BMJ Support Palliat Care.

[8] Kirk D, Kabdebo I, Whitehead L (2021). Prevalence of distress, its associated factors and referral to support services in people with cancer. J Clin Nurs.

[9] Duan Y, Wang L, Sun Q, Liu X, Ding S, Cheng Q, Xie J, Cheng ASK (2021). Prevalence and determinants of psychological distress in adolescent and young adult patients with cancer: a multicenter survey. Asia Pac J Oncol Nurs.

[10] Garvey G, Cunningham J, Janda M, Yf He V, Valery PC (2018). Psychological distress among Indigenous Australian cancer survivors. Support Care Cancer.

[11] Lee HH, Chiu CC, Lin JJ, Wang JJ, Lee KT, Sun DP, Shi HY (2019). Impact of preoperative anxiety and depression on quality of life before and after resection of hepatocellular carcinoma. J Affect Disord.

[12] Phoosuwan N, Lundberg PC (2022). Psychological distress and health-related quality of life among women with breast cancer: a descriptive cross-sectional study. Support Care Cancer.

[13] Bi Z, Li W, Zhao J, Pang L, Jing Y, Zhang X, Yao S, Yin X, Zuo H, Cheng H (2022). Negative correlations of psychological distress with quality of life and immunotherapy efficacy in patients with advanced NSCLC. Am J Cancer Res.

[14] Velure GK, Muller B, Hauken MA (2022). Symptom burden, psychological distress, and health-related quality of life in cancer survivors with pelvic late radiation tissue injuries. Support Care Cancer.

[15] Liu YJ, Schandl A, Markar S, Johar A, Lagergren P (2021) Psychological distress and health-related quality of life up to 2 years after oesophageal cancer surgery: nationwide population-based study. BJS Open 5. 10.1093/bjsopen/zraa038 10.1093/bjsopen/zraa038 PMC7893460 33609371

[16] Ebob-Anya BA, Bassah N (2022). Psychosocial distress and the quality of life of cancer patients in two health facilities in Cameroon. BMC Palliat Care.

[17] Karunanithi G, Sagar RP, Joy A, Vedasoundaram P (2018). Assessment of psychological distress and its effect on quality of life and social functioning in cancer patients. Indian J Palliat Care.

[18] Verma R, Balhara YP, Gupta CS (2011). Gender differences in stress response: role of developmental and biological determinants. Ind Psychiatry J.

[19] Chaplin TM (2015). Gender and emotion expression: a developmental contextual perspective. Emot Rev.

[20] Zhang Y, Zhang H, Song L, Tang L (2010). Application of the NCCN distress thermometer in Chinese cancer patients. Chinese Men Health J.

[21] Thapa S, Sun H, Pokhrel G, Wang B, Dahal S, Yu S (2020). Performance of distress thermometer and associated factors of psychological distress among Chinese cancer patients. J Oncol.

[22] Cella DF, Tulsky DS, Gray G, Sarafian B, Linn E, Bonomi A, Silberman M, Yellen SB, Winicour P, Brannon J (1993). The Functional Assessment of Cancer Therapy scale: development and validation of the general measure. J Clin Oncol.

[23] Wan C, Meng Q, Tang X, Zhang C, Luo J, Zhang X (2006). Evaluation of the Chinese version of the Cancer Patient Quality of Life Measurement Scale FACT-G. J Pract Oncol.

[24] Zhao H, Li X, Zhou C, Wu Y, Chen L (2021). Psychological distress among Chinese patients with breast cancer undergoing chemotherapy: concordance between patient and family caregiver reports. J Adv Nurs.

[25] Hong J, Wei Z, Wang W (2015). Preoperative psychological distress, coping and quality of life in Chinese patients with newly diagnosed gastric cancer. J Clin Nurs.

[26] Tian X, Jin Y, Chen H, Tang L, Jiménez-Herrera MF (2021). Relationships among social support, coping style, perceived stress, and psychological distress in Chinese lung cancer patients. Asia Pac J Oncol Nurs.

[27] Zabora J, BrintzenhofeSzoc K, Curbow B, Hooker C, Piantadosi S (2001). The prevalence of psychological distress by cancer site. Psycho-oncology.

[28] Henselmans I, Helgeson VS, Seltman H, de Vries J, Sanderman R, Ranchor AV (2010). Identification and prediction of distress trajectories in the first year after a breast cancer diagnosis. Health Psychol.

[29] Kim SJ, Rha SY, Song SK, Namkoong K, Chung HC, Yoon SH, Kim GM, Kim KR (2011). Prevalence and associated factors of psychological distress among Korean cancer patients. Gen Hosp Psychiatry.

[30] Aminisani N, Nikbakht H-A, Shojaie L, Jafari E, Shamshirgaran M (2021). Gender differences in psychological distress in patients with colorectal cancer and its correlates in the Northeast of Iran. J Gastrointest Cancer.

[31] Swash B, Bramwell R, Hulbert-Williams NJ (2017). Unmet psychosocial supportive care needs and psychological distress in haematological cancer survivors: the moderating role of psychological flexibility. J Contextual Behav Sci.

[32] Pacheco-Barcia V, Gomez D, Obispo B, MihicGongora L, Hernandez San Gil R, Cruz-Castellanos P, Gil-Raga M, Villalba V, Ghanem I, Jimenez-Fonseca P, Calderon C (2022). Role of sex on psychological distress, quality of life, and coping of patients with advanced colorectal and non-colorectal cancer. World J Gastrointest Oncol.

[33] Donato KM, León-Pérez G, Wallston KA, Kripalani S (2018). Something old, something new: when gender matters in the relationship between social support and health. J Health Soc Behav.

[34] Alsubaie MM, Stain HJ, Webster LAD, Wadman R (2019). The role of sources of social support on depression and quality of life for university students. Int J Adolesc Youth.

[35] Cai A, Lou Y, Long Q, Yuan J (2016). The sex differences in regulating unpleasant emotion by expressive suppression: extraversion matters. Front Psychol.

[36] Iimura S, Taku K (2018). Gender differences in relationship between resilience and big five personality traits in Japanese adolescents. Psychol Rep.

[37] Ivan S, Daniela O, Jaroslava BD (2023). Sex differences matter: males and females are equal but not the same. Physiol Behav.

[38] James KA, Stromin JI, Steenkamp N, Combrinck MI (2023). Understanding the relationships between physiological and psychosocial stress, cortisol and cognition. Front Endocrinol.

[39] Martínez-Mesa J, González-Chica DA, Duquia RP, Bonamigo RR, Bastos JL (2016). Sampling: how to select participants in my research study?. An Bras Dermatol.

